# AGING INTERVENTIONS GET HUMAN: CAN WE EXTEND HEALTHSPAN?

**DOI:** 10.1093/geroni/igz038.2989

**Published:** 2019-11-08

**Authors:** Brian Kennedy

**Affiliations:** National University of Singapore, Not applicable, Singapore

## Abstract

Understanding biologic aging will afford opportunities for novel interventions to enhance human healthspan. If ageing can be slowed, the effect would be simultaneous protection from many of the chronic diseases. One strategy is to use animal model organisms to find common pathways that modulate ageing and then to seek methods for their human manipulation. The TOR pathway is one point of convergence and a clinically approved drug targeting the TOR kinase, rapamycin, extends murine lifespan and healthspan. Many more small molecules are being added to the list of anti-ageing compounds. Here, I use examples of interventions to conceptualize how agents extending healthspan might improve human health. We are entering a stage in aging research where it is imperative to test ageing interventions in humans and several strategies are contemplated. The potential to directly impact human healthspan is emerging from ageing research and this approach, if successful, will have global impact.

